# Improving nutritional assessment in GLP-1RA therapy: Beyond self-reports to equitable interventions

**DOI:** 10.1016/j.obpill.2025.100213

**Published:** 2025-09-30

**Authors:** Parth Aphale, Himanshu Shekhar, Shashank Dokania

**Affiliations:** Dr. D.Y. Patil Vidyapeeth (Deemed to be University), Pimpri, Pune, Maharashtra, India

Dear Editor,

We read with great interest the article by Johnson et al. (2025) exploring diet quality and nutrient distribution among individuals using GLP-1 receptor agonists (GLP-1RAs) for weight management. The study offers timely insight into dietary patterns in this rapidly expanding therapeutic population. However, several methodological and interpretive aspects warrant further discussion to contextualize the findings and refine future directions [1].

First, while the Healthy Eating Index (HEI) provides a robust measure of diet quality, it was originally developed for general population diets rather than pharmacologically reduced caloric intake. GLP-1RA users often consume markedly lower volumes of food, making nutrient density and adequacy per calorie more clinically relevant than absolute HEI scores. A nutrient adequacy ratio framework could complement HEI to better assess whether reduced energy intake still supports micronutrient sufficiency, as deficiencies have been frequently reported in obesity management settings [2].

Second, the study highlights skewed protein intake concentrated at dinner. Yet, beyond distribution, the quality of protein plant versus animal sources plays a critical role in long-term metabolic outcomes. Given the underrepresentation of seafood and plant proteins in this cohort, it would be useful to investigate whether targeted nutrition counseling emphasizing diverse protein sources could mitigate lean mass loss, a key concern with rapid weight reduction [3].

Third, the reliance on self-reported 3-day food records, while acknowledged, introduces potential recall and desirability biases. Wearable-based dietary monitoring or digital photographic methods are emerging as more objective tools and may reduce under-reporting [4]. Integrating such approaches in future studies could substantially enhance accuracy.

Moreover, cultural and socioeconomic dimensions of food choice deserve deeper exploration. The current sample was predominantly White and highly educated, raising concerns about generalizability. GLP-1RA uptake shows disparities across racial and socioeconomic groups [5]. Dietary quality in more diverse cohorts may differ substantially, influenced by food affordability, access, and cultural preferences. Addressing these contextual factors will be crucial for designing equitable dietary guidance.

Finally, while the authors propose dietary counseling for GLP-1RA users, the potential synergy of meal-timing interventions such as early time-restricted eating remains underexplored. Meta-analyses suggest front-loading calories earlier in the day yields improvements in glycemic control and weight outcomes. Such strategies could be particularly beneficial given the observed concentration of caloric intake at dinner in this cohort.

In conclusion, Johnson et al. make an important contribution by highlighting the suboptimal diet quality among GLP-1RA users. Future studies should incorporate nutrient adequacy ratios, objective dietary assessment tools, culturally diverse populations, and structured interventions integrating protein distribution and meal timing. These refinements will better inform practical, personalized nutrition guidance in the context of pharmacological weight management.

## CRediT author statement

**HS**- Conception and Design, Final review, Conceptualization. **PA**- Data acquisition, Writing the manuscript. **Shashank Dokania:** Analysis, Interpretation.

## Declaration of generative AI

The authors declare that AI-based language assistance was used solely for grammar refinement. All intellectual content, interpretation, and conclusions are the sole responsibility of the authors.

## Funding and disclosures

None.

## Declaration of competing interest

The authors declare that they have no known competing financial interests or personal relationships that could have appeared to influence the work reported in this paper.
